# A toxic subset of soluble α-synuclein species in dementia with Lewy body

**DOI:** 10.1093/braincomms/fcaa016

**Published:** 2020-03-24

**Authors:** Diana L Castillo-Carranza

**Affiliations:** Health Science Department, Medical School, Universidad de Monterrey, Monterrey, Mexico

## Abstract

This scientific commentary refers to ‘Analysis of α-synuclein species enriched from cerebral cortex of humans with sporadic dementia with Lewy bodies’, by Sanderson *et al.* (https://doi.org/10.1093/braincomms/fcaa010).

**This scientific commentary refers to ‘Analysis of α-synuclein species enriched from cerebral cortex of humans with sporadic dementia with Lewy bodies’, by Sanderson *et al.* (** https://doi.org/10.1093/braincomms/fcaa010).

For decades researchers have sought to find cures for health conditions that affect the brain (as imagined by the artist Peter Rogers in Fig. 1). Dementia with Lewy body (DLB) and Parkinson’s disease are two of the several brain diseases, collectively called synucleinopathies. They comprise a group of chronic neurological disorders characterized by intracellular inclusions of α-synuclein (αSyn) and the progressive and irreversible neurodegeneration. The physiological effects of these diseases do not manifest until mid-to-late adulthood. Similar to other dementias, there is no cure for DLB. The insidious symptoms of DLB are vague, and the initial changes that may drive αSyn dysfunction are completely unknown (McKeith *et al.*, 2017).

**Figure 1:**
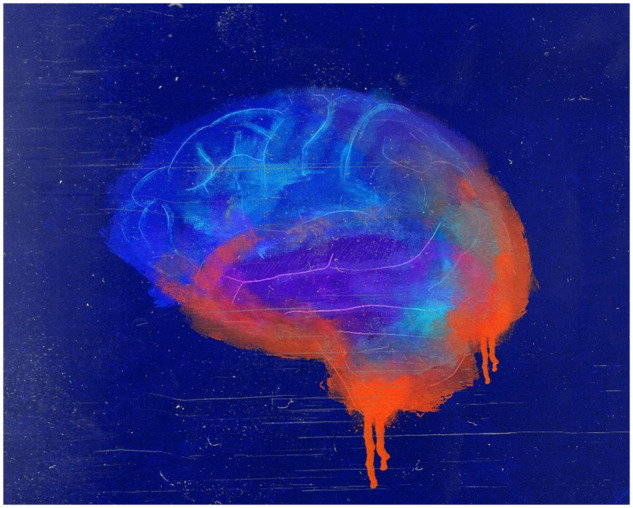
**The brain under attack.** ** Image reproduced by permission of the artist. Copyright Peter Rogers.**

In this issue, Sanderson *et al.* (2020) took a step towards unveiling dysfunctional αSyn contributing to the sporadic DLB pathology, by providing compelling evidence of the existence of neurotoxic soluble αSyn species in the human brain. This small protein (140 amino acids), which is abundant in presynaptic terminals in a healthy brain, is the main component of Lewy body deposits and Lewy neurites in synucleinopathies. Like other amyloidogenic proteins, αSyn has structural characteristics that make it prone to aggregate; the protein is arranged into three distinct domains that likely contribute to its aggregation and deposition. For instance, familial forms of Parkinson’s disease are associated with mutations in the N-terminal domain of αSyn. In addition, crystal structure analysis revealed that residues 68–78 and 47–56 from the central core are prone to form β-sheets, which are characteristics of amyloid assemblies. Furthermore, C-terminally truncated forms of αSyn appeared to aggregate faster than the full-length protein. These observations suggest that the different regions of the protein somehow aggregate compromising cell function and survival.

In the DLB brain, as well as Parkinson’s disease, the regional staging of αSyn deposits suggests a spatiotemporal progression of the pathology. The transfer of dysfunctional αSyn between interconnected neurons predicts the pattern of Lewy pathology and neurodegeneration in Parkinson’s disease (Del Tredici and Braak, 2020). The first direct evidence of αSyn spreading in the human brain has come from the development of Lewy pathology in grafts of patients with Parkinson’s disease receiving foetal human midbrain neurons (Hansen *et al.*, 2011). Nevertheless, these large aggregates do not appear to be the main cause of neuronal deterioration. For instance, amyloid plaques considered for long time as the main toxic structure in Alzheimer’s disease do not correlate with cognitive decline and neurodegeneration. These large extracellular deposits can be even found in normally aging people, thus questioning the role of amyloid plaques in Alzheimer’s disease. Similarly, in Alzheimer’s disease and other neurodegenerative diseases, post-mortem analysis of older individuals revealed extensive Lewy pathology with no evident Parkinson-like symptoms. All of these findings point to a different conformation that might be responsible for neuronal impairment.

It is now well accepted based on a strong body of evidence that oligomers, rather than the large fibrils, are likely to be involved in neurodegeneration. The αSyn oligomers are small soluble toxic intermediates, which are en route to fibrils formation and recently shown to be the end product of the aggregation process *in vitro* (Breydo and Uversky, 2015). These unstable, recombinant and/or brain-derived oligomers have shown to be toxic when applied extracellularly to the cells in culture or injected into the mouse brain. The dynamic properties, structural diversity and limited suitable methods to analyse oligomers represent a challenge. Only a handful of studies have provided evidence of these soluble oligomers in the human brain. A key challenge in the field that we need to overcome on the road to develop effective therapeutics is to develop a more thorough picture of which oligomeric forms of αSyn are toxic and how they confer this toxicity. Sanderson *et al.* (2020) have helped address this challenge by examining αSyn oligomers isolated from human disease tissue and their toxic effects in human induced pluripotent stem cell-derived neurons.

The work from Sanderson *et al.* (2020) broadens previous work from others and provides evidence for the presence of soluble neurotoxic αSyn aggregates in DLB brain tissue. As previously shown, αSyn species can be detected using specific tools designed to recognize these unstable intermediates. As such, the use of conformational-dependent antibodies has proven to be valuable to identify oligomers in synucleinopathies (Sengupta *et al.*, 2015; Castillo-Carranza *et al.*, 2018).

Following a different approach, Sanderson *et al.* (2020) developed a method to investigate αSyn species in a cohort of patients with a neuropathological diagnosis of DLB and also control brains, by using a sequence of non-denaturing biochemical fractionation followed by enzyme-linked immunosorbent assay (ELISA) analysis. They measured absolute αSyn by ELISA and found enriched αSyn species in the cytosolic fraction of the DLB cortex, which was lower in the membrane-associated fraction. Apparently, the levels of αSyn in the cytosolic fraction corresponded with the degree of Lewy neuropathology. The subcellular redistribution of membrane-bound αSyn to the cytosolic compartment coincided with a recent report showing the abundance of vesicle-bound αSyn in Lewy bodies (Burre *et al.*, 2012.; Shahmoradian *et al.*, 2019). In other words, the shift of αSyn from the membrane to cytosol may suggest a loss of αSyn physiological function. This is a critical protein required for the survival of distinct neuronal populations, which is also involved in the compartmentalization, storage, and release of synaptic vesicles, required for neurotransmission. If accurate, these findings may support the hypothesis that αSyn loses its function in the DLB cortex.

Sanderson *et al.* (2020) then analysed the soluble fraction using non-denaturing size-exclusion chromatography fractionation followed by ELISA quantitation and found an increase in the soluble high molecular weight αSyn species (∼2 MDa to 440 kDa). Although soluble high molecular weight αSyn aggregates were found in both control and DLB cortex, permeabilization assays showed that only αSyn species from DLB cases destabilized lipid membranes compared with the control brains. Their findings suggested that soluble αSyn aggregates obtained from DLB brains exert cytotoxic effects by permeabilizing membranes. These findings are consistent with the concept that soluble intermediates display toxic properties. Oligomers are known to vary in size (dimers, trimers, multimers), shape (granular, globulomers, annular) and β-sheet content (Breydo and Uversky, 2015); thus, it is possible that one or more distinct αSyn species are responsible for the effects observed here.

Going beyond the soluble αSyn species, the authors investigated the effects of sonicated detergent-insoluble αSyn on human induced pluripotent stem cell neurons. They determined the impact of insoluble αSyn by measuring morphological changes inflicted to the neurites. Although the effects of insoluble αSyn to induced pluripotent stem cells are clear, recent work has proven that sonication of recombinant fibrils from distinct amyloidogenic protein can break into short fibrils and oligomers among other conformations (Ghag *et al.*, 2018), thus representing a source of distinct species. Given that the protein aggregation is a dynamic process, this could suggest that the toxic effects observed here may be linked to the oligomeric foci.

The work from Sanderson *et al.* (2020) provides important information regarding αSyn species involved in DLB while introducing a new perspective in respect to the distribution of pathology in the brain tissues used in this work. The microheterogeneity within the same tissue represents a confounding factor that should be taken into consideration, thus highlighting the need for a better sampling in research.

The search for clues that will help to understand the pathology of the disease and the earlier brain changes in DLB before the symptoms develop is still underway. Without a doubt, the techniques described by Sanderson *et al.*, in this issue of *Brain Communications*, provided valuable information to better understand the contribution of αSyn species to DLB pathology and related synucleinopathies.
